# Mixed stock analysis of juvenile green turtles aggregating at two foraging grounds in Fiji reveals major contribution from the American Samoa Management Unit

**DOI:** 10.1038/s41598-019-39475-w

**Published:** 2019-02-28

**Authors:** Susanna Piovano, Aisake Batibasaga, Ana Ciriyawa, Erin L. LaCasella, Peter H. Dutton

**Affiliations:** 1 School of Marine Studies, The University of the South Pacific, Suva, Fiji; 2 Fiji Ministry of Fisheries, Suva, Fiji; 3 NOAA Fisheries Southwest Fisheries Science Center, La Jolla, CA USA

## Abstract

In this study we assessed the breeding population, or Management Unit (MU), origin of green turtles (*Chelonia mydas*) present at Yadua Island and Makogai Island foraging grounds in Fiji, central South Pacific. Based on analysis of mitochondrial (mt) DNA sequences from 150 immature green turtles caught during surveys carried out in 2015–2016, we identified a total of 18 haplotypes, the most common being CmP22.1 (44%) which is a primary haplotype characterizing the American Samoa breeding population. Results of a Bayesian mixed-stock analysis reveals that the two foraging grounds are used by green turtles from the American Samoa MU (72%, Credible Interval (CI): 56–87%), New Caledonia MU (17%, CI: 6–26%) and French Polynesia MU (7%, CI: 0–23%). The prominence of the contribution we found from the American Samoa MU compared to that of French Polynesia, both which have historic telemetry and tagging data showing connectivity with Fijian foraging areas, may reflect the current relative abundance of these two nesting populations and draws attention to a need to update population surveys and identify any significant nesting in Fiji that may have been overlooked.

## Introduction

The green turtle *Chelonia mydas* has a circumglobal distribution ranging from tropical to warm temperate waters^1^. In tropical waters, juvenile green turtles undergo an ontogenetic shift from an omnivorous diet in the pelagic environment to a primarily herbivorous diet in the shallow waters of coastal neritic environments^2,3^ when about 3–10 years old^4,5^. Green turtles reach maturity at 15–42 years^5^ and have a non-annual reproductive cycle; adult females exhibit natal homing and return to lay their eggs on the sandy beaches of their natal region^1^ with a remigration interval (non-nesting phase) of about 3 years^6^, while adult males show fidelity to courtship areas^7^ and have a non-breeding phase of about 1–2 years^8^. Green turtles of the same rookery may use different foraging grounds^9^, and a single individual can visit and exploit different foraging grounds^10^. Mixed aggregations from various rookeries have also been reported^9^.

**Figure 1:**
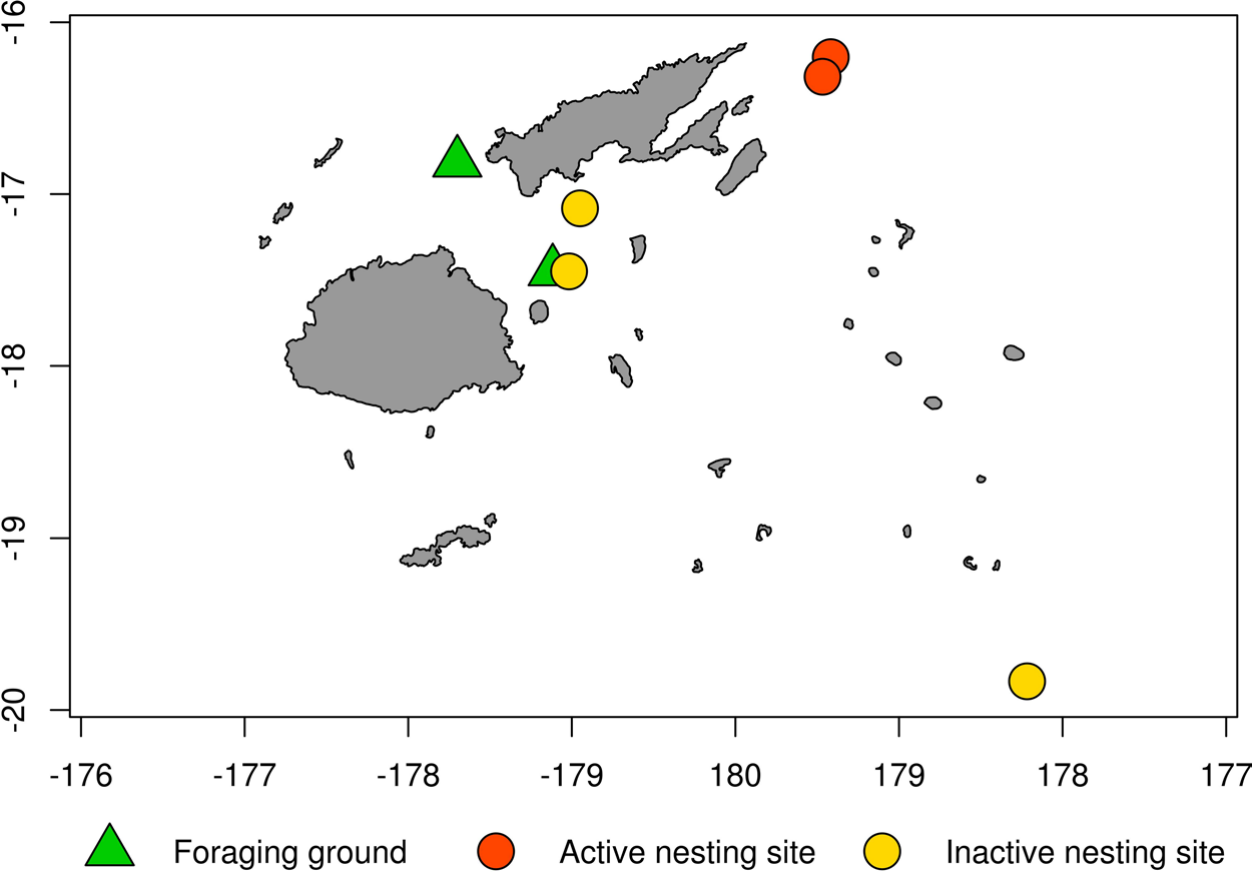
Green turtle foraging grounds surveyed in this study (Yadua Island 16.823S, 178.284W and Makogai Island 17.450S, 178.948W) and known green turtle nesting sites (Heemskercq Reef 16.283S, 179.467E and the Ringgold Reef 16.317S, 179.467E systems are currently active, while Makogai Island, Namena-lala Island 17.083 S, 179.050W and Vatoa Island 19.833S, 178.217E are no longer in use), Fiji, central South Pacific. Map created with R^57^ with packages “map” and “mapdata”.

Green turtles nest and forage in Fiji, in the central South Pacific (Fig. 1). While there have not been comprehensive green turtle nesting surveys that adequately cover the entire region of Fiji, which is made up of more than 300 islands and 500 islets, effort to identify nesting sites over the last few decades have failed to reveal significant nesting, with a small number of nests recorded in a few localities^11–17^. The majority of the islands surveyed by Hirth^18^ and Guinea^14^ had no sign of green turtle nesting activity. However, four green turtles nesting sites, Makogai Island, Namena-lala Island, Heemskercq Reef and the Ringgold Reef systems have been identified and surveyed at least once every ten years in the last four decades^11,14–17^. The number of green turtle nests has been consistently low at Heemskercq Reef and the Ringgold Reef systems (average number of green turtle nests observed per survey <10^11,14,16,17^). Green turtle nests have no longer been found at Makogai Island and Namena-lala since the 1980s^15^. The green turtle nesting colony on Vatoa Island, in the south of Fiji, reportedly disappeared in the late 1970s^13^. The Fiji nesting population of green turtles has been estimated in about 50–75 adult females^16^ based on unpublished data from year 2000 and a market survey done in 1996^19^. Despite the sparsity of observed nesting in Fiji^20^, the green turtle is considered to be the most abundant of the four sea turtles species regularly seen in Fiji waters^14^.

Genetic characterization of mitochondrial (mt) DNA of the green turtle population nesting in Fiji is not known. Mitochondrial DNA has been extensively used to delineate breeding population structure into management units (MUs) which, in turn, can be used in mixed-stock analysis (MSA) to characterize the genetic composition and natal origins of sea turtle foraging aggregations^21^. These studies have provided insights into connectivity between foraging grounds used by resident green turtles and their natal breeding areas (rookeries) in the eastern, central North and Indo-Pacific^22–30^. However, there is a lack of information from genetic studies for the western and central South Pacific Ocean islands^31^.

Both adult and immature green turtles forage in Fiji. Use of Fijian foraging grounds by adult males and by females nesting in other Pacific countries has been demonstrated by tagging studies and satellite telemetry carried out between 1972 and 2010^1,13,20^. A total of 19 adult females were found (mark-recapture) or tracked (satellite telemetry) to Fiji during post-reproductive migration. Of these, eight individuals were nesters from American Samoa^1,32^, five from French Polynesia^1,14,20,33^, three from Australia (one reported by Jit^34^, the other two are from Colin Limpus pers. comm.), two from Tonga^35^, and one in the Cook Islands^1^. Connectivity with adult males from French Polynesia waters was also suggested by the migration to Fiji of three individuals released from pens and tagged on Scilly Atoll^1,36^. Additional insights on the origin of immature and adult green turtles foraging in Fiji were provided by a market survey carried out in Suva, Fiji’s capital, in 1996. Results from a pilot study using genetic analysis of green turtles sampled from market stalls suggested additional possible links to rookeries in the northern Great Barrier Reef and Micronesia^19^. In general, origin of immature green turtles and the contribution of each regional rookery to Fijian foraging aggregations remain unknown.

The aim of this study was to assess the stock composition of green turtle aggregations at Yadua Island and Makogai Island foraging grounds by using molecular markers (mtDNA) on samples from wild ranging individuals captured and released *in situ*.

## Results

A total of 150 immature green turtles were sampled during six surveys in Yadua (n = 91, CCL: mean 52.1 cm, range: 25.5–74.5 cm) and four surveys in Makogai (n = 59, CCL: mean 57.9 cm, range: 43.5–97.0 cm).

There was a total of 18 haplotypes identified within the two study areas (Table 1). Seventeen haplotypes were observed in Yadua and nine in Makogai. CmP33.1 was the only haplotype found in Makogai that was not found in Yadua. CmP22.1 was the most common haplotype at both sites, identified in 44% of the samples analysed. CmP65.1 was the second most common haplotype observed in 22%, followed by CmP44.1 in 8.6% of the sample set. CmP20.1 was found in 5.3% of the samples in equal proportions between the two sites. There were five newly described haplotypes, in 4.0% of the samples, which have not been identified in any MU to date (known as orphan haplotypes) (CmP33.1, CmP239.1-CmP242.1; Genbank IDs: MK471335-MK471339). A sixth orphan haplotype (CmP192.1), found in 2.7% of the samples (Table 1) has been previously described at a foraging ground in Australia, but to date not identified at any rookery^25^. The remaining haplotypes were found in low frequencies among both sites (Table 1). There was no difference in haplotype frequencies between the Yadua and Makogai (P > 0.05), so the samples were combined for the MSA.

**Table 1:** Haplotypes identified in the aggregation of green turtles (n = 150) in the foraging grounds of Yadua Island (Vanua Levu) and Makogai Island (Lomaiviti), Fiji.

Haplotype	Yadua Island	Makogai Island
CmP20.1	4	4
CmP22.1	41	25
CmP31.1	4	1
CmP33.1	0	1
CmP44.1	6	7
CmP47.1	2	2
CmP56.1	1	0
CmP57.2	1	0
CmP65.1	16	17
CmP65.2	1	1
CmP80.1	4	0
CmP85.1	2	0
CmP160.1	1	0
CmP192.1	3	1
CmP239.1	1	0
CmP240.1	1	0
CmP241.1	2	0
CmP242.1	1	0
Total	91	59

**Figure 2:**
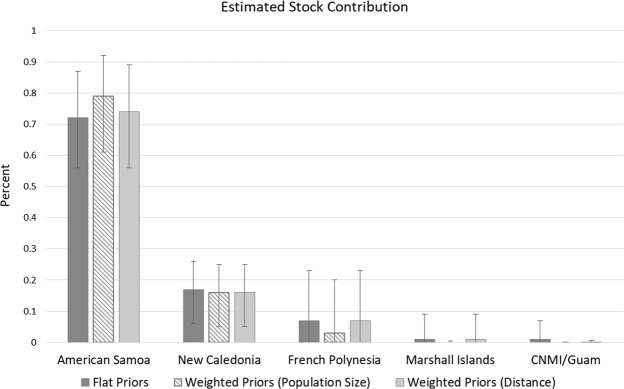
Estimated mixed-stock analysis (MSA) stock contributions from *Chelonia mydas* Management Units (MUs) throughout the Pacific. Of the 25 potential source rookeries, only the highest contributors (>1%) are shown. Mean and credible intervals (95%) are shown.

The MSA with uniform priors indicated that the majority of green turtles foraging in the two foraging grounds were composed of animals from the American Samoa MU (72%; Credible Interval (CI): 56–87%). New Caledonia was estimated to contribute 17% (CI: 6–26%) and French Polynesia 7% (CI: 0–23%) (Fig. 2). The combined contributions from the Commonwealth of the Northern Mariana Islands (CNMI)/Guam and Marshall Islands MUs were minimal with an estimated mean of 1%, and CI spanning zero. In general, results were similar for weighted priors, with distance having a slightly less effect than population size (Fig. 2). Taking population size into account reduced the contribution from French Polynesia and eliminated Marshall Islands and CNMI/Guam, allocating a greater contribution from American Samoa (Fig. 2), however all point estimates had overlapping CIs. Potential contributions from other Pacific locations were inconclusive and therefore unlikely sources.

## Discussion

Our findings that the Fijian foraging aggregations in our study comprised immature green turtles from American Samoa and French Polynesia, taken together with previous telemetry studies showing post-nesting migration of nesters from American Samoa and French Polynesia to Fiji^1,37^, highlight the relevance of Fijian foraging grounds for these two MUs. The contribution we detected from New Caledonia MU represents a new finding that adds to the complex connectivity among green turtle rookeries and foraging areas in the Pacific Islands, which extends outside the central South Pacific to the western South Pacific. The similarity of results for the MSA weighted by population size and distance and those using uniform priors suggest that the estimates based on our genetic data are relatively robust, as indicated also by the similar CI for all three models. Even though the New Caledonia population is thought to be orders of magnitude larger than the American Samoa^38^, the weighted model estimates barely change for New Caledonia, and increase slightly for American Samoa.

Overall, both immature (this study) and mature^1,14,20,32,35^ green turtles from the central South Pacific and western South Pacific use seagrass meadows and algal beds in Fiji. However, the lack of large individuals in our survey suggests that they may do so in separate aggregations. For example, none of the adult females that travelled to Fiji after being equipped with a satellite transmitter at their nesting sites reached waters around Yadua Island or Makogai Island^32,34^. Seagrass meadows are present in the shallow coastal waters around the two main islands of Fiji (Viti Levu and Vanua Levu)^39^ and several of the small island groups (Yasawa, Mamanuca, Lomaiviti and Lau)^16,39^, however the extent of seagrass area coverage is unknown^40,41^. Information on sea turtles foraging at some of these other seagrass areas has been limited to occasional reports^11,14,18^ as well as single-individual tracking^32^ so it is not possible to assess the overall size and distribution of the Fijian foraging aggregations of green turtles. Mapping presence and area coverage of seagrass meadows together with their use by green turtles, would be a useful baseline to understand green turtle populations’ ecology and dynamics in this largely unexplored area of the central South Pacific.

We found Yadua had twice the number of haplotypes detected at Makogai, however this is likely a consequence of the larger sample size obtained in the first site, rather than an indication of greater haplotype diversity, especially given that there was no significant difference in haplotype frequencies. Additional sampling is warranted to explore this further.

The majority of the green turtles we sampled were from the American Samoa MU. Rose Atoll, the major nesting area in American Samoa^38^, is about 1400 km north-east from the study sites and it is an uninhabited National Wildlife Refuge administered jointly by the U.S. Fish and Wildlife Service and the American Samoa Government. The site is also included in Rose Atoll Marine National Monument and part of the American Samoa’s Whale and Turtle Sanctuary. The size of the breeding population has been estimated at 105 breeding females^38^, however this may be an underestimation due to the nesting sites being remote, largely unsurveyed and the breeding population might be potentially recovering (Mark MacDonald pers. comm.).

Our estimates of 6–26% (mean 17%) contribution from the New Caledonia MU, located about 1700 km west of Fiji is of interest given the lack of connectivity evident from previous tagging and telemetry studies in adult nesters. A recent review of 50 years of tagging data at the main nesting site, D’Entrecasteaux atolls, shows that the majority of the New Caledonian green turtle nesters forage in the Great Barrier Reef in Australia, while a smaller percentage remain in New Caledonia’s waters^42^. However, it is worth noting that a recent genetic MSA study found that the immature green turtles at one of New Caledonia’s largest foraging ground, Gran Lagon Sud, primarily belonged to the New Caledonia MU^24^. The reviewed studies however, do not necessarily track dispersal of hatchlings from nesting beaches. Our results suggest that a portion of the population of immature green turtles from New Caledonia may be transported eastward periodically by eddies that feed into the South Pacific Gyre, despite the prevailing westward flow of the South Equatorial current that passes New Caledonia (see Read *et al*.^24^). Fine-scale ocean current modelling would be useful to test this hypothesis (see Naro-Maciel *et al*.^28^).

Scilly Atoll in French Polynesia, located about 3000 km east of Fiji, was historically the largest green turtle nesting site of the central South Pacific, with an estimated 1050 breeding females^38^ (based on 1991 data), but contributed only a small percentage (7%) to the aggregations at Yadua Island and Makogai Island foraging grounds. There is relatively high uncertainty around the mean estimated contribution from French Polynesia from our MSA as indicated by a CI of 0–23%. This likely stems from the low sample size for this MU in the baseline dataset^31^ and the presence of CmP65.1 in both American Samoa and French Polynesia which would have resulted in uncertainty in assignments between these MUs, possibly underestimating the relative contribution of French Polynesia. Nevertheless, even at the upper bound of the CI (23%) the estimate for French Polynesia is well below that of the lower bound of the CI for American Samoa (56%) (Fig. 2). Although our sample was limited to immature individuals, based on data from nesting females’ movements from early 1970s to early 2010s^14,33,36^, a higher contribution from the French Polynesia MU was expected. Based on satellite telemetry, the average home range of adult females resident in Fiji was about 27 km^2^^32^, so the low estimated number of immature green turtles from this MU might result from a different distribution of the French Polynesian turtles in other Fijian seagrass meadows. However, these results might also reflect a decline for the French Polynesia population^43^. With average age at maturity estimated at approximately 29 years for green turtles (range 15–42 years in Avens and Snover^5^ for different populations and aging methods), none of the immature individuals we sampled were born when data for the currently available estimates of nesting female population size were collected. The relatively low proportion we estimated as a contribution from the French Polynesia MU might be the result of a lower relative abundance from the dramatic population decline that appears to have occurred in recent decades coupled with the closer proximity of American Samoa to Fiji. It is worth noting that green turtles from French Polynesia made up an estimated 37% of the green turtles sampled at Suva fish markets in 1996–1997^19^. While the genetic pilot study referred to in Boyle^19^ did not include baseline data from several key rookeries, and the turtle harvest reported by Boyle^19^ did not come from our study sites, this intense and sustained harvest of immature green turtles in Fiji may have played a part in reducing the number of individuals reaching adulthood and migrating back to French Polynesia to reproduce. Exploration of other Fiji foraging grounds is necessary to ascertain whether there are spatial or temporal patterns that may reflect historic patterns of recruitment. At present, we can only make inferences based upon the two foraging areas that were investigated in this study, therefore additional information from other coastal waters may change the picture.

Having representative samples from all the potential nesting populations is required for a more accurate MSA (see Komoroske *et al*.^21^), which underscores the need for extended surveys of Fiji’s remote islands to assess presence of green turtle nesting and collect samples for genetic analysis. Larger representative sample sizes from nesting sites in French Polynesia and American Samoa will also help improve accuracy of future MSA. The presence of orphan haplotypes in our Fiji sample set illustrates the need for larger sample sizes, and may represent haplotypes that are present in Fiji nesting populations that have been overlooked. The relatively low frequencies of each of the orphan haplotypes generally indicates presence of rare haplotypes in populations that have been under-sampled, and that are detected once larger sample sizes are obtained (see Komoroske *et al*.^21^). Our results do however suggest that the American Samoa nesting population is much larger than current knowledge, or alternatively, that there is significant nesting somewhere in Fiji that has been overlooked.

We did not find any haplotypes from the eastern Pacific rookeries in Fiji which, while rare, have been detected at foraging areas off New Zealand^44^ and in waters around the Oceanic Islands (Hawaii and Palmyra Atoll) in the North Pacific^22,28^. Also lacking from the mixed stock aggregations were individuals from northern Great Barrier Reef and Micronesia, whose presence in Fiji was suggested in the 20 year old pilot study reported by Boyle^19^ from the market survey but for which no size range of harvested turtles was provided. Finally, the elimination of CNMI/Guam as a source based on the population size weighted MSA model combined with the CI spanning zero in both weighted and uniform prior MSA results is consistent with the small nesting population.

Based on our findings, we suggest monitoring of Yadua Island and Makogai Island foraging grounds to ascertain temporal variation in stock composition and demographic (size-classes) structure, and exploration of other foraging grounds to determine presence of mature green turtles. This type of analysis would greatly benefit from the mtDNA characterization of the local green turtle rookery. The prominence of the contribution we found from the American Samoa MU compared to that of French Polynesia, both which have historic telemetry and tagging data showing connectivity with Fijian foraging areas, may reflect the current relative abundance of these two nesting populations and draws attention to a need to update population surveys for the French Polynesia MU, whose relatively high population estimates are based on old information from a 1991–1995 survey^38^.

This MSA study is the first to be carried out in the waters of Fiji and provides insight into the foraging habits of immature green sea turtles in the region. The patterns found in this study are restricted to two sites (that together represent less than 1% of total Fiji coastline) and should not be extended to foraging aggregations throughout the country’s coastal waters. Nevertheless, the contributions of the American Samoa, New Caledonia and French Polynesia MUs that we have found, further emphasize the connection between these four Pacific Island Countries and highlight the importance of looking beyond territorial waters when planning population management of green turtles in the region.

## Methods

This study was carried out at two green turtle foraging grounds: Yadua Island (16.823S, 178.284W) on the West of Vanua Levu and Makogai Island (17.450S, 178.948W) in the Lomaiviti group. The survey extended along a total of 54 km of coastline; 38 km at Yadua Island and 16 km at Makogai Island. The total length of coastline in Fiji is estimated at 6123 km (all coastline lengths estimated from Fiji Ministry of Lands and Mineral Resources, Department of Lands, 1:50,000 scale GIS data of Fiji coastline). Seagrass meadows at the two study sites have a patchy distribution and occurred from the intertidal to the subtidal zones, to a maximum depth of 4 m. The two sites, totalling 10 km^2^ (surveyed surface was 7 km^2^ at Yadua Island and 3 km^2^ at Makogai Island), are located about 100 km apart at the opposite entrances of the Vatu-i-Ra Channel, where the Bligh Water Current flows from South-East to North-West (Fig. 1).

Green turtles were surveyed using capture, mark (flipper-tagging) and recapture, during a pilot study in July and August 2015, and then from November 2015 to July 2016. Each survey lasted four consecutive days, during which sea turtles were hand captured from a small boat^45^ during daylight at high tide. At first capture of each individual, biological data (species ID, curved carapace length (CCL), weight and sex when possible) were recorded and two skin samples collected by using a biopsy punch (2 mm diameter on small immatures and 6 mm diameter on large immatures), as described in Dutton *et al*.^46^. With the exception of three individuals previously tagged by Fiji Fisheries Department, all turtles were marked on the right front flipper^20^ with a titanium tag for long term identification.

### Genetic analysis

Genomic DNA was extracted from epidermal tissue samples using a sodium chloride protocol described by Miller *et al*.^47^. Primers LCM15382 and H950g^48^ were used to amplify an ~889 bp fragment at the 5′ end of the control region of the mitochondrial genome using polymerase chain reaction (PCR) methodologies^49^. PCR products were sequenced with an Applied Biosystems® model 3730 automated DNA sequencer (Applied Biosystems, Foster City, CA, USA). Haplotypes were assigned by comparing aligned sequences against a local reference library of published and unpublished green turtle haplotype sequences using Geneious version 8 (Biomatters) as well as searching the database on GenBank (http://www.ncbi.nlm.nih.gov). The haplotype composition and frequency of our sampled Fiji foraging aggregation (as the stock mix) was compared with those published for MUs (as potential sources) across the Pacific^23–25,31,50–54^. We estimated stock composition of the Fiji foraging index site by conducting MSA using the program BAYES^55^. Only 25 MUs that had any haplotypes also present in our Fiji foraging study were included as potential source MUs for the MSA and haplotypes not reported at any MU were excluded in the MSA. A total of 100,000 Markov Chain Monte Carlo steps were run for four independent chains, each with different starting points. A burn-in of 50,000 runs was used to calculate the posterior distribution. The Gelman and Rubin shrink factor diagnostic was calculated as part of the BAYES analysis to test for convergence of all chains^55^. The MSA was run using uniform priors, where potential source MUs were weighted equally, and two models that weighted priors by relative population size and distance from the foraging ground respectively. The weighted models assume that larger MUs are more likely to contribute to the mixed stock than smaller ones (population size priors) or alternatively that MUs in closer proximity to the foraging grounds are more likely to contribute than distant ones, and can help address uncertainty arising from haplotypes that are shared among multiple MUs, as long as the assumption is correct and biologically meaningful^25^. The size of each nesting population was based on numbers summarized in Ng *et al*.^27^ and FitzSimmons and Limpus^56^ (Supplementary Table S1). Distances between the midpoint of our two foraging sites and each MU, or its centroid in case of multiple rookeries, were measured as straight lines with ImageJ (http://rsb.info.nih.gov/ij) on the map from Jensen *et al*.^25^ (Supplementary Table S1).

This research was performed in accordance with relevant guidelines and regulations, and in compliance with the 2004–2018 Fiji “Moratorium on molesting, taking or killing of turtles”. Sampling procedures were approved by the “Animal Ethics Committee” section of The University of the South Pacific (USP) Research Committee.

## Supplementary information

Supplementary Table
